# Can ChatGPT 4.0 Diagnose Epilepsy? A Study on Artificial Intelligence’s Diagnostic Capabilities

**DOI:** 10.3390/jcm14020322

**Published:** 2025-01-07

**Authors:** Francesco Brigo, Serena Broggi, Eleonora Leuci, Gianni Turcato, Arian Zaboli

**Affiliations:** 1 Innovation, Research and Teaching Service (SABES-ASDAA), Teaching Hospital of the Paracelsus Medical Private University (PMU), 39100 Bolzano, Italy; arian.zaboli@sabes.it; 2 Neurology and Stroke Unit, ASST dei Sette Laghi, 21100 Varese, Italy; 3 Division of Neurology, “Franz Tappeiner” Hospital, 39012 Merano, Italy; 4 Department of Internal Medicine, Intermediate Care Unit, Hospital Alto Vicentino (AULSS-7), 36014 Santorso, Italy

**Keywords:** artificial intelligence, diagnosis, epilepsy, large language models

## Abstract

**Objectives**: This study investigates the potential of artificial intelligence (AI), specifically large language models (LLMs) like ChatGPT, to enhance decision support in diagnosing epilepsy. AI tools can improve diagnostic accuracy, efficiency, and decision-making speed. The aim of this study was to compare the level of agreement in epilepsy diagnosis between human experts (epileptologists) and AI (ChatGPT), using the 2014 International League Against Epilepsy (ILAE) criteria, and to identify potential predictors of diagnostic errors made by ChatGPT. **Methods**: A retrospective analysis was conducted on data from 597 patients who visited the emergency department for either a first epileptic seizure or a recurrence. Diagnoses made by experienced epileptologists were compared with those made by ChatGPT 4.0, which was trained on the 2014 ILAE epilepsy definition. The agreement between human and AI diagnoses was assessed using Cohen’s kappa statistic. Sensitivity and specificity were compared using 2 × 2 contingency tables, and multivariate analyses were performed to identify variables associated with diagnostic errors. **Results**: Neurologists diagnosed epilepsy in 216 patients (36.2%), while ChatGPT diagnosed it in 109 patients (18.2%). The agreement between neurologists and ChatGPT was very low, with a Cohen’s kappa value of −0.01 (95% confidence intervals, CI: −0.08 to 0.06). ChatGPT’s sensitivity was 17.6% (95% CI: 14.5–20.6), specificity was 81.4% (95% CI: 78.2–84.5), positive predictive value was 34.8% (95% CI: 31.0–38.6), and negative predictive value was 63.5% (95% CI: 59.6–67.4). ChatGPT made diagnostic errors in 41.7% of the cases, with errors more frequent in older patients and those with specific medical conditions. The correct classification was associated with acute symptomatic seizures of unknown etiology. **Conclusions**: ChatGPT 4.0 does not reach human clinicians’ performance in diagnosing epilepsy, showing poor performance in identifying epilepsy but better at recognizing non-epileptic cases. The overall concordance between human clinicians and AI is extremely low. Further research is needed to improve the diagnostic accuracy of ChatGPT and other LLMs.

## 1. Introduction

In 2005, a Task Force of the International League Against Epilepsy (ILAE) defined epilepsy as “a disorder of the brain characterized by an enduring predisposition to generate epileptic seizures, along with the neurobiological, cognitive, psychological, and social consequences of this condition” [1]. This definition specifies that at least one epileptic seizure is required for the diagnosis of epilepsy. In 2014, the ILAE refined this definition by introducing a more practical framework. According to the revised definition, epilepsy can be diagnosed under the following conditions: (1) at least two unprovoked (or reflex) seizures occurring more than 24 h apart, as in the previous definitions, or (2) when an epilepsy syndrome is identified. Notably, epilepsy can also be diagnosed after a single unprovoked (or reflex) seizure if the risk of recurrence is estimated to be 60% or higher over the next 10 years [2]. This marked a shift from earlier definitions, which required a minimum of two unprovoked seizures occurring 24 h apart for a diagnosis. The seizures must be unprovoked (or reflexes), meaning they should occur without any obvious precipitating factors or conditions [2].

The 2014 ILAE definition is grounded in data on the likelihood of seizure recurrence following two unprovoked seizures. One study estimated the risk of recurrence at 73% (95% CI: 59–87) over the subsequent four years [3]. A 60% threshold for diagnosis was set based on the lower limit of the confidence interval for this recurrence risk. However, in daily clinical practice, it is not always straightforward to determine whether the risk of seizure recurrence after the first unprovoked seizure meets or exceeds 60% over the next 10 years. This challenge complicates the practical application of the definition of epilepsy. While certain etiologies, such as stroke, are often associated with a high risk of recurrence [4], the risk estimates for other causes, such as brain trauma or cerebral infection, remain imprecise and are not always linked to a high recurrence risk [5,6].

Recently, there has been growing interest in the use of artificial intelligence (AI), particularly large language models (LLMs) like ChatGPT [7], as tools to enhance decision support in diagnosis and risk stratification [8,9]. AI tools have the potential to improve diagnostic accuracy and support physicians by increasing efficiency, reducing time and resource use, and facilitating rapid decision-making. Since the diagnosis of epilepsy involves a probabilistic assessment of seizure recurrence risk, there is speculation about whether a widely popular LLM-based system like ChatGPT, if specifically trained on the 2014 ILAE epilepsy definition, could effectively assist in diagnosing epilepsy.

To explore this, we designed a study aimed at comparing the level of agreement in epilepsy diagnosis between human experts (epileptologists) and AI (ChatGPT) using the practical criteria outlined by the ILAE in 2014. Hence, we evaluated the performance of diagnosing epilepsy in people with epileptic seizure(s) based on the current ILAE definition. Additionally, we sought to identify potential predictors of diagnostic errors made by ChatGPT, which could guide future improvements in the tool’s diagnostic capabilities.

## 2. Materials and Methods

**Figure 1:**
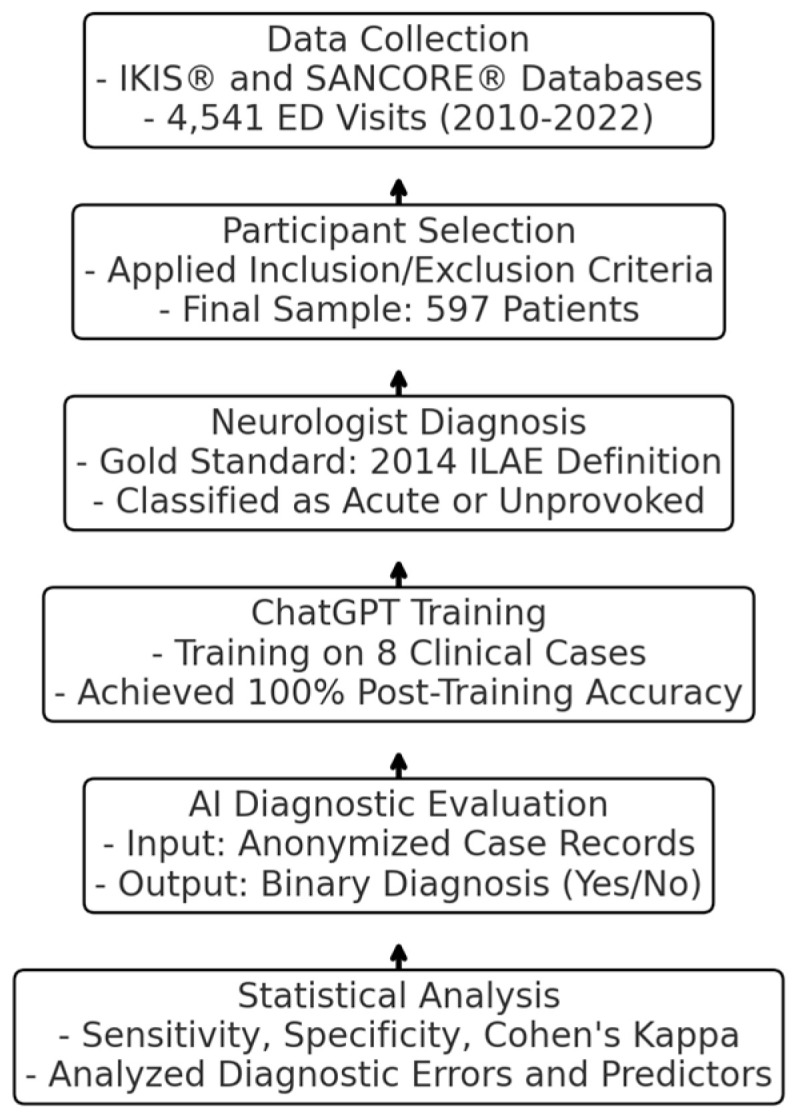
Schematic Representation of Study Design.

A schematic of the study design showing the main stages of the study, with a precise description of the procedure for each stage, is reported in Figure 1.

### 2.1. Study Design and Setting

This retrospective, observational, single-center study was conducted using a database of consecutive emergency department (ED) visits at Merano Hospital (Bolzano, Italy), aiming to identify patients presenting with either a first epileptic seizure or the first recurrence of a previously diagnosed seizure. The Manchester Triage System is used in this ED for the risk stratification of incoming patients.

In South Tyrol, the health data of every patient who had had at least one visit or investigation at any of the seven hospitals in the Autonomous Province of Bolzano were recorded in an electronic health record system (IKIS^®^). These data are automatically integrated into a centralized digital database (SANCORE^®^), which compiles information from ED visits, outpatient consultations, diagnostic investigations, and hospitalizations.

For ED visits, the available data included triage forms, medical and nursing history, vital signs, blood test results, diagnostic investigations, specialist consultation reports, administered therapies, and discharge details.

### 2.2. Participants

#### 2.2.1. Database Screening and Manual Review

A random sample was extracted from the database of consecutive ED visits at Merano Hospital between 1 January 2010, and 30 June 2022.

The database was sorted by individual identification codes, and specific search filters were applied to isolate visits related to epileptic seizures or other paroxysmal and transient events (e.g., syncope, loss of consciousness, hypertensive crises, and paroxysmal vertigo). To create a random sample from the larger dataset, a new column was added, and random numbers were generated for each row. The dataset was then sorted based on these random numbers. A total of 4541 ED visits were identified, and each visit was manually screened to identify patients presenting with a first epileptic seizure or the first recurrence of an epileptic seizure.

#### 2.2.2. Inclusion Criteria

Inclusion criteria were as follows: (a) an ED visit for the first epileptic seizure or (b) an ED visit for the first recurrence of an epileptic seizure, provided that the date and characteristics of the initial seizure were clearly documented. This was important to determine when the first diagnosis of epilepsy could be made, in line with the 2014 ILAE definition, which allows for epilepsy diagnosis after either a first unprovoked (or reflex) seizure with a high risk of recurrence or after the first recurrence of an unprovoked seizure, provided the initial event occurred more than 24 h earlier [2].

#### 2.2.3. Exclusion Criteria

The exclusion criteria were as follows: (a) patients who were tourists or non-residents of South Tyrol due to insufficient follow-up information; (b) ED visits unrelated to epileptic seizures or their recurrence; (c) ED visits for non-epileptic paroxysmal events, as determined by a neurologist or electronic health record review; (d) visits for the first recurrence of an epileptic seizure where the date of the initial seizure could not be verified; and (e) visits for second or subsequent recurrences in patients with already diagnosed epilepsy.

#### 2.2.4. Data Collection

For each identified patient, the complete set of health data from the electronic health record was reviewed by two neurologists with three years of experience in epilepsy (S.B. and E. L.) to collect relevant information. Each epileptic seizure was classified as either acute symptomatic or unprovoked [10]. Only patients whose seizures could be clearly classified into these categories were included in this study. Furthermore, it was assessed whether cases with a first epileptic seizure or the first recurrence of an epileptic seizure could be diagnosed with epilepsy according to the 2014 ILAE official definition [2]. Classification decisions were based on patient history obtained by ED nurses during triage, the ED physician’s medical reports, the results of any neuroimaging investigations or EEG performed in the ED (if available), and the neurologist’s assessment (if available). Unclear cases, as well as cases involving seizures due to progressive conditions, such as neurodegenerative diseases or tumors, were excluded due to ambiguity in classification, as these have been referred to as progressive symptomatic seizures by some Authors. Classification decisions and inclusion/exclusion determinations were made by the two neurologists, with disagreements resolved through consultation with a senior neurologist with 14 years of epilepsy expertise (F.B.). Patient information obtained after discharge from the ED, such as long-term video-EEG recordings or neuroimaging investigations, was not included in the assessment of epilepsy diagnosis according to the 2014 ILAE definition.

For each patient who met the inclusion criteria, data were extracted on variables including age, sex, type of seizure (acute symptomatic or unprovoked), and etiology (structural, genetic, infectious, metabolic, immune, or unknown). Additional data included whether the epileptic event was status epilepticus, the presence of impaired consciousness (e.g., somnolence, stupor, coma) at ED admission, the occurrence of tongue biting or sphincter release, and relevant comorbidities such as hypertension, ischemic heart disease, chronic heart failure, diabetes, or prior stroke. The full text of patient complaints from triage assessments, medical history recorded by ED physicians, and results of neuroimaging investigations were also included. All data were organized into a predefined form using Excel (Microsoft^®^).

### 2.3. AI Training and Utilization

ChatGPT 4.0 (OpenAI) was provided with the necessary information to diagnose epilepsy according to the ILAE definitions of 2005 and 2014 by being given access to the official reference documents [1,2].

Subsequently, ChatGPT was trained using eight case examples from the 2014 ILAE definition, simulating real-life decision-making scenarios. These cases were input into the system, and ChatGPT was tasked with diagnosing epilepsy. Specifically, we asked ChatGPT whether each presented case fulfilled the 2014 ILAE definition of epilepsy. In instances of incorrect diagnosis, structured training was conducted under the guidance of an expert neurologist specializing in epilepsy, who provided information that ChatGPT had missed. After this training process, the eight clinical cases were re-submitted, and ChatGPT achieved a 100% correct diagnosis rate.

At this stage, ChatGPT was deemed ready to evaluate clinical cases. The clinical cases selected from the retrospective database, related to ED visits for a first epileptic seizure or the first recurrence of an epileptic seizure, were formatted in a structured manner. This included a personal ID, patient history obtained by ED nurses during triage, and the ED physician’s medical reports, including details on neurological examination, semiology of epileptic seizures, and blood tests whenever available. Information on relevant comorbidities, such as hypertension, ischemic heart disease, chronic heart failure, diabetes, or prior stroke, was provided. Additionally, where available, reports of neuroimaging investigations and EEG performed in the ED were included. Neuroimaging data were presented not as images but as written reports by the radiologists. Information from EEG was obtained using recording conditions and durations on a case-by-case basis. All information was delivered in a fully anonymized form, excluding any parameters that were considered sensitive data.

Upon receiving the clinical cases, ChatGPT was asked to make a binary diagnosis of epilepsy (Yes or No) based on the provided documents and its prior training, adhering to the 2014 ILAE criteria. Hence, we inquired if ChatGPT could determine whether each case met the 2014 ILAE definition of epilepsy.

### 2.4. Statistical Analyses

To assess the accuracy of ChatGPT’s epilepsy diagnosis compared to that of neurologists, a 2 × 2 contingency table was created, with the neurologists’ evaluation considered the gold standard. From this table, the sensitivity, specificity, positive predictive value (PPV), and negative predictive value (NPV) were calculated along with their respective 95% confidence intervals (95% CI). The agreement between ChatGPT and the neurologists was assessed using an unweighted Cohen’s kappa, reported with a 95% CI.

#### 2.4.1. Error Analysis

To identify patient characteristics associated with diagnostic errors, a univariate analysis was performed. Patients were divided into two groups: those correctly classified by ChatGPT (in agreement with neurologists) and those incorrectly classified by ChatGPT (in disagreement with neurologists). All collected variables were analyzed. Continuous variables were described using the mean and standard deviation (SD) for normally distributed data or the median and interquartile range (IQR) for non-normally distributed data. Categorical variables were reported as absolute numbers and percentages. Comparisons between groups for continuous variables were performed using the Student’s *t*-test, Mann-Whitney U test, or Kruskal-Wallis test, as appropriate, while the Chi-square test was used for categorical variables.

#### 2.4.2. Multivariable Analysis

Subsequently, multivariable analysis was conducted using logistic regression to evaluate which variables were independently associated with diagnostic errors. Results were reported as Odds Ratios (OR) with their respective 95% CIs. All analyses were considered statistically significant with a *p*-value < 0.05, and were conducted using STATA 16.1.

#### 2.4.3. Sample Size Calculation

Since this was an exploratory study aimed at generating hypotheses or preliminary data, we did not perform a formal statistical power calculation to identify trends and potential predictors of diagnostic errors.

### 2.5. Ethical Considerations

The study received approval from the Local Ethics Committee (approval number: 49.2024; date of approval: 26 March 2024) and was conducted in accordance with the Declaration of Helsinki, adhering to ethical principles for medical research involving human subjects.

## 3. Results

### 3.1. Overview of Study Participants

A total of 597 patients were included in this study. According to an evaluation conducted by neurologists, 216 patients (36.2%) were diagnosed with epilepsy, while ChatGPT diagnosed epilepsy in 109 patients (18.2%) (Table 1).

### 3.2. Diagnostic Agreement Between ChatGPT and Neurologists

The agreement between the neurologists and ChatGPT in diagnosing epilepsy was assessed using unweighted Cohen’s kappa, which yielded a value of −0.01 (95% CI: −0.08 to 0.06), indicating no significant agreement.

### 3.3. Diagnostic Performance of ChatGPT

ChatGPT’s sensitivity in diagnosing epilepsy was 17.6% (95% CI: 14.5–20.6), with a specificity of 81.4% (95% CI: 78.2–84.5). The PPV was 34.8% (95% CI: 31.0–38.6), while the NPV was 63.5% (95% CI: 59.6–67.4).

### 3.4. Analysis of Diagnostic Errors

ChatGPT made an error in diagnosing epilepsy in 41.7% (249/597) of the cases. The clinical characteristics associated with these diagnostic errors are summarized in Table 2. Compared to patients correctly classified by ChatGPT, those in whom diagnostic errors occurred were generally older, more likely to have status epilepticus, and had higher rates of ischemic heart disease and prior strokes as comorbidities. Additionally, patients with diagnostic errors had fewer acute symptomatic seizures.

Notably, errors were more frequent in patients with genetic or structural etiologies for their seizures, while they were less common in those with metabolic, infectious, or unknown causes.

### 3.5. Factors Associated with Correct Classification

The only two clinical variables significantly associated with correct classification by ChatGPT were acute symptomatic seizures (OR, 0.050; 95% CI, 0.024–0.102) and unknown etiology (OR, 0.066; 95% CI, 0.023–0.188).

## 4. Discussion

This study is the first to evaluate the performance of an LLM system trained to diagnose epilepsy in real clinical cases using the official 2014 ILAE definition. It shows that ChatGPT 4.0 significantly underperforms compared to human experts in diagnosing epilepsy. These findings highlight the need for further refinement of AI models to improve diagnostic accuracy, as ChatGPT currently does not achieve epileptologist performance in diagnosing epilepsy.

Preliminary evidence suggests that AI could be helpful in identifying interictal epileptiform discharges [11,12,13,14,15] and neuroimaging abnormalities associated with epilepsy [16,17,18]. Conversely, while ChatGPT and DALL·E 2, another AI tool, can identify and visually interpret the burden of epilepsy from medical, societal, and economic perspectives, their ability to engage in in-depth discussions of epilepsy remains limited [19]. However, the lack of research on the objective use of AI in diagnosing epilepsy, based on official definitions, underscores a significant gap in the literature. This highlights the urgent need for further studies to validate and enhance the diagnostic capabilities of AI tools for epilepsy and other neurological disorders. The present study could serve as a foundation for future advancements and improvements in AI-driven medical diagnostics, which could in turn, enhance the performance of ChatGPT’s responses to questions related to epilepsy [20,21].

The degree of concordance between human experts and artificial intelligence (AI) in epilepsy diagnosis was extremely low, as demonstrated by the negative values in the unweighted Cohen’s kappa analysis. This suggests that the observed agreement between ChatGPT and the epileptologists was less than what would be expected by chance alone. ChatGPT’s sensitivity in diagnosing epilepsy was very low, indicating a poor ability to correctly identify patients with epilepsy. However, its specificity was relatively high, suggesting that ChatGPT had a better ability to correctly classify patients who did not meet the criteria for epilepsy.

Patients correctly classified by ChatGPT were more likely to have had acute symptomatic seizures, which was also independently associated with a correct diagnosis. Acute symptomatic seizures are not included in the definition of epilepsy, as they are considered an acute reaction of the brain to injury or a systemic disorder that temporarily lowers the seizure threshold [22]. Once the precipitating factor or condition is resolved, the seizures are not expected to recur. In cases of brain injury, such as stroke, the timing between the injury and the seizure is critical [2,4,5,10]. Seizures that occur beyond the acute phase are more likely to result from structural changes in the brain, leading to a persistent predisposition to seizures or epilepsy. A typical example of acute symptomatic seizures includes those due to alcohol withdrawal or metabolic disturbances like electrolyte imbalances, where the risk of subsequent unprovoked seizures is very low [10,22]. Notably, ChatGPT correctly classified most patients with seizures of metabolic origin (69/80, 86%), suggesting some capacity for recognizing conditions associated with a low risk of recurrence.

Conversely, ChatGPT performed poorly in correctly identifying patients with epilepsy, as diagnosed by epileptologists. The model relied on the full text of patient complaints during triage, medical histories recorded by ED physicians, and neuroimaging results. It is possible that ChatGPT incorporated some of the initial assessments or diagnostic impressions made by triage nurses or ED physicians, which may have been influenced by patient comorbidities. For example, it is possible that medical histories recorded by nurses or physicians placed undue emphasis on comorbid conditions, such as previous stroke or ischemic heart disease, which are associated with acute symptom onset (e.g., sudden neurological deficits or acute myocardial infarction) and could be mistaken for epileptic seizures. This could explain the higher prevalence of ischemic heart disease and previous stroke among patients in whom ChatGPT made a diagnostic error.

Seizure etiology also varied between patients correctly and incorrectly classified by ChatGPT in terms of epilepsy diagnosis. However, only an unknown etiology was independently associated with the correct assessment by ChatGPT. This suggests that in cases where clear data on etiology were lacking, ChatGPT may have taken a more conservative approach, refraining from making an epilepsy diagnosis. Conversely, structural etiologies were more frequent among patients in whom ChatGPT made diagnostic errors. This may reflect bias in the initial diagnostic judgment or medical documentation, but it also suggests that ChatGPT struggled to account for the time interval between a brain injury and the first epileptic seizure, which is crucial for distinguishing between acute symptomatic and unprovoked seizures. Additionally, the higher prevalence of previous stroke among patients incorrectly classified by ChatGPT indicates that the model was likely unable to adequately link epileptic seizures to this etiology and classify them as unprovoked.

Our retrospective study has some limitations. This study was conducted at a single center in an ED setting, which may limit the generalizability of the findings. However, this setting reflects everyday clinical practice, as most patients with a first epileptic seizure (and likely those with a first recurrence) are likely to seek medical assistance at the ED. The decision to include only patients with a first-ever epileptic seizure or first recurrence was intended to replicate the clinical scenario where a diagnosis of epilepsy can be made, in accordance with the 2014 ILAE definition, which allows for a diagnosis either after a first unprovoked (or reflex) seizure with a high recurrence risk or after the first recurrence, provided that the first event occurred more than 24 h earlier. The study did not include all patients presenting with a first epileptic seizure or recurrence during the study period; however, a large random sample was selected to minimize selection bias. Furthermore, instead of focusing on seizure semiology, we provide details regarding the classification of seizures as acute symptomatic or unprovoked when reporting data. This approach reflects the different risks of recurrence associated with these seizure types, which significantly impacts the diagnosis. Similarly, we have included details on the etiology of seizures, as this can affect the diagnosis of epilepsy. However, for cases with a structural cause for their seizure, due to a lack of information, we could not specify the localization and size of the structural brain defects for every included patient. Neuroimaging data were provided to both epileptologists and ChatGPT in the form of written reports by radiologists rather than images. This decision was made to ensure a fair and balanced comparison, preventing any advantage in diagnostic accuracy for either party.

Training ChatGPT on only eight cases of epilepsy and subsequently evaluating its diagnostic performance might initially seem insufficient. While it is likely that training on a larger dataset could improve ChatGPT’s classification accuracy by better exposing it to the variability and nuances of epilepsy diagnoses, we chose not to train the model on a broader range of cases for two reasons. Firstly, we wanted to train ChatGPT 4.0 based on the cases provided in the official ILAE definition, as these cases were selected by the authors for their emblematic nature. Secondly, physicians are theoretically expected to rely on official documents that provide diagnostic guidance, such as those used for training ChatGPT. Conversely, if we had trained ChatGPT to diagnose epilepsy according to the official ILAE definition using a larger dataset, we could have placed ChatGPT in a position of superiority over its human counterparts, potentially disadvantaging them. Although it is possible that the limited number of cases used for training contributed to ChatGPT’s suboptimal performance, it is important to consider that, ideally, the official definition of epilepsy should, in itself, be necessary and sufficient for adoption in clinical practice. However, this may not be the case in reality. The authors of the ILAE 2014 paper aimed to facilitate the diagnosis of epilepsy in cases of a single epileptic seizure by requiring a 60% probability of recurrence [2]. They did not, however, provide detailed guidance on how to calculate this percentage, given the variability of clinical cases. Therefore, it is possible that the official definition alone may not be sufficient to diagnose epilepsy using LLMs. Further training and additional studies are necessary to improve diagnostic accuracy. Another limitation of this study is that ChatGPT was restricted to a binary Yes or No classification. This constraint may have contributed to its underperformance, particularly in cases where a definitive epilepsy diagnosis was not feasible. While the same binary classification was applied to the epileptologists, they had the advantage of resolving disagreements by consulting a third opinion, a process not available to ChatGPT. This difference in the resolution of ambiguous cases could have impacted the comparative diagnostic accuracy.

The eight case scenarios from the ILAE 2014 document used for training ChatGPT, while illustrative and emblematic, include newly introduced terms like “resolved epilepsy” and explore the boundaries of “probable or possible epilepsy.” The limited number of cases used as examples may not capture the full spectrum of epilepsy cases, potentially leading to insufficient training and poor diagnostic accuracy of ChatGPT.

Both ChatGPT and epileptologists had access to the same clinical data, including patient complaints, medical histories, and neuroimaging results. However, epileptologists were able to apply their knowledge and skills to interpret clinical data within a structured diagnostic framework, relying on focused history-taking and the results of neurological examinations. This difference in approach is an intrinsic and unavoidable limitation of this study. While ChatGPT was provided with information from triage assessments and medical histories, these records may have been incomplete or collected differently by nurses and physicians, which could have affected its performance. However, given that in Italy, triage and ED medical records are official documents, it is unlikely that critical clinical details were omitted or inadequately documented.

The results of this study are dependent on ChatGPT’s configuration at the time, which is subject to frequent updates. Hence, future studies, even those adopting the same methodology, may yield different results. It is also possible that ChatGPT’s performance would have been different using texts written in English.

Since this was an exploratory study designed to generate hypotheses and preliminary data, we did not perform a formal statistical power calculation. However, with a sample size of 597 patients, our study benefited from a relatively large dataset, which enhanced the reliability and generalizability of our findings. This substantial sample size also provides a solid foundation for future replication studies.

Given the preliminary nature of this study, we did not assess ChatGPT’s performance separately for first seizures and recurrent seizures, as doing so would have reduced the model’s statistical power and overall performance. Instead, we evaluated its accuracy in diagnosing epilepsy more broadly. Unfortunately, we did not record the proportion of cases that required the opinion of a third epileptologist.

Despite these limitations, our study applied ChatGPT after it had been trained using the official ILAE criteria for epilepsy diagnosis, and its performance was tested on a large sample of real-world cases.

## 5. Conclusions

This study demonstrates that ChatGPT 4.0 does not reach human clinicians’ ability to accurately diagnose epilepsy in patients presenting to the ED with either a first-ever seizure or the first recurrence of a seizure. Despite being trained with the official 2014 ILAE definition of epilepsy, ChatGPT 4.0 currently shows very poor performance in correctly identifying patients with epilepsy. However, it performs better at recognizing patients who do not meet the criteria for epilepsy diagnosis. Nonetheless, the overall level of concordance between human clinicians and artificial intelligence remains extremely low, with the agreement being worse than random chance.

Looking forward, the rapid development of AI in medical diagnostics offers promising avenues for future research. Improving training datasets, introducing more diverse clinical scenarios, and working closely with medical professionals may enhance diagnostic accuracy. Future research should investigate whether ChatGPT and other large language models can be refined and integrated with additional diagnostic modalities to deliver more reliable and comprehensive decision support for epilepsy diagnosis.

## Figures and Tables

**Table 1:** A 2 × 2 contingency table showing the results of the epilepsy diagnosis assessment made by epileptologists compared to that made by ChatGPT. The values of sensitivity, specificity, positive predictive value (PPV), and negative predictive value (NPV) are also provided.

Epilepsy Diagnosis Assessment by ChatGPT (Artificial Intelligence)	Epilepsy Diagnosis Assessment by Epileptologists (Human Intelligence)	
	Epilepsy No	Epilepsy Yes
Epilepsy No	310	178
Epilepsy Yes	71	38
Sensitivity: 17.6% (95% CI 14.5–20.6)		
Specificity: 81.4% (95% CI 78.2–84.5)		
PPV: 34.8% (95% CI 31.0–38.6)		
NPV: 63.5% (95% CI 59.6–67.4)		

**Table 2:** Clinical characteristics associated with errors in epilepsy diagnosis made by ChatGPT.

Variables	Overall, 597 Patients (100.0%)	Correct Epilepsy Diagnosis Assessment by ChatGPT, 348 Patients (58.3%)	Error Epilepsy Diagnosis Assessment by ChatGPT, 249 Patients (41.7%)	*p*-Value
Sex, n (%)				0.837
Male	341 (57.1)	200 (57.5)	141 (56.6)	
Female	256 (42.8)	148 (42.5)	108 (43.4)	
Age, median (IQR)	54 (22–74)	51 (23–71)	56 (22–71)	0.046
Seizure type, n (%)				<0.001
Acute symptomatic	223 (37.3)	179 (51.4)	44 (17.7)	
Unprovoked	374 (62.7)	169 (48.6)	205 (82.3)	
Seizure etiology, n (%)				<0.001
Structural	222 (37.2)	72 (20.7)	150 (60.2)	
Genetic	45 (7.5)	10 (2.9)	35 (14.1)	
Infectious	85 (14.2)	61 (17.5)	24 (9.6)	
Metabolic	80 (13.4)	69 (19.8)	11 (4.4)	
Immune	7 (1.2)	5 (1.4)	2 (0.8)	
Unknown	158 (26.5)	131 (37.6)	27 (10.8)	
Status epilepticus, n (%)	48 (8.9)	21 (6.7)	27 (12.0)	0.034
Consciousness status in the ED				0.213
Awake and responsive	453 (75.9)	269 (77.3)	184 (73.9)	
Somnolence/stupor	114 (19.1)	59 (16.9)	55 (22.1)	
Coma	30 (5.0)	20 (5.8)	10 (4.0)	
Sphincter release, n (%)	42 (7.0)	27 (7.7)	15 (6.0)	0.414
Tongue biting, n (%)	98 (16.4)	56 (16.1)	42 (16.9)	0.801
Comorbidities				
Hypertension	199 (33.3)	107 (30.7)	92 (36.9)	0.113
Ischemic heart disease	49 (8.2)	20 (5.7)	29 (11.6)	0.010
Chronic heart failure	41 (6.9)	19 (5.5)	22 (8.8)	0.108
Diabetes	48 (8.0)	32 (9.2)	16 (6.4)	0.220
Previous stroke	111 (18.6)	38 (10.9)	73 (29.3)	<0.001

## Data Availability

Data are available to researchers upon reasonable request.

## References

[1] Fisher R.S., van Emde Boas W., Blume W., Elger C., Genton P., Lee P., Engel J. (2005). Epileptic seizures and epilepsy: Definitions proposed by the International League Against Epilepsy (ILAE) and the International Bureau for Epilepsy (IBE). Epilepsia.

[2] Fisher R.S., Acevedo C., Arzimanoglou A., Bogacz A., Cross J.H., Elger C.E., Engel J., Forsgren L., French J.A., Glynn M. (2014). ILAE official report: A practical clinical definition of epilepsy. Epilepsia.

[3] Hauser W.A., Rich S.S., Lee J.R., Annegers J.F., Anderson V.E. (1998). Risk of recurrent seizures after two unprovoked seizures. N. Engl. J. Med..

[4] Hesdorffer D.C., Benn E.K., Cascino G.D., Hauser W.A. (2009). Is a first acute symptomatic seizure epilepsy? Mortality and risk for recurrent seizure. Epilepsia.

[5] Zelano J. (2021). Recurrence risk after a first remote symptomatic seizure in adults: Epilepsy or not?. Epilepsia Open.

[6] Brigo F., Zelano J., Abraira L., Bentes C., Ekdahl C.T., Lattanzi S., Lossius M.I., Redfors P., Rouhl R.P.W., Russo E. (2024). Proceedings of the “International Congress on Structural Epilepsy & Symptomatic Seizures” (STESS, Gothenburg, Sweden, 29–31 March 2023). Epilepsy Behav..

[7] OpenAI ChatGPT (Mar 14 Version) [Large Language Model]. https://chat.openai.com/chat.

[8] Thirunavukarasu A.J., Ting D.S.J., Elangovan K., Gutierrez L., Tan T.F., Ting D.S.W. (2023). Large language models in medicine. Nat. Med..

[9] Zaboli A., Brigo F., Sibilio S., Mian M., Turcato G. (2024). Human intelligence versus Chat-GPT: Who performs better in correctly classifying patients in triage?. Am. J. Emerg. Med..

[10] Beghi E., Carpio A., Forsgren L., Hesdorffer D.C., Malmgren K., Sander J.W., Tomson T., Hauser W.A. (2010). Recommendation for a definition of acute symptomatic seizure. Epilepsia.

[11] Kural M.A., Jing J., Fürbass F., Perko H., Qerama E., Johnsen B., Fuchs S., Westover M.B., Beniczky S. (2022). Accurate identification of EEG recordings with interictal epileptiform discharges using a hybrid approach: Artificial intelligence supervised by human experts. Epilepsia.

[12] Tveit J., Aurlien H., Plis S., Calhoun V.D., Tatum W.O., Schomer D.L., Arntsen V., Cox F., Fahoum F., Gallentine W.B. (2023). Automated Interpretation of Clinical Electroencephalograms Using Artificial Intelligence. JAMA Neurol..

[13] Kleen J.K., Guterman E.L. (2023). The New Era of Automated Electroencephalogram Interpretation. JAMA Neurol..

[14] King-Stephens D. (2024). AI and EEG: Should EEGers RIP (Rest in Peace)?. Epilepsy Curr..

[15] Mansilla D., Tveit J., Aurlien H., Avigdor T., Ros-Castello V., Ho A., Abdallah C., Gotman J., Beniczky S., Frauscher B. (2024). Generalizability of electroencephalographic interpretation using artificial intelligence: An external validation study. Epilepsia.

[16] Chang A.J., Roth R., Bougioukli E., Ruber T., Keller S.S., Drane D.L., Gross R.E., Welsh J., Abrol A., Calhoun V. (2023). MRI-based deep learning can discriminate between temporal lobe epilepsy, Alzheimer’s disease, and healthy controls. Commun. Med..

[17] Lee D.A., Ko J., Kim H.C., Shin K.J., Park B.S., Kim I.H., Park J.H., Park S., Park K.M. (2021). Identifying juvenile myoclonic epilepsy via diffusion tensor imaging using machine learning analysis. J. Clin. Neurosci. Off. J. Neurosurg. Soc. Australas..

[18] Kerr W.T., McFarlane K.N. (2023). Machine Learning and Artificial Intelligence Applications to Epilepsy: A Review for the Practicing Epileptologist. Curr. Neurol. Neurosci. Rep..

[19] Puteikis K., Mameniškienė R. (2024). Artificial intelligence: Can it help us better grasp the idea of epilepsy? An exploratory dialogue with ChatGPT and DALL·E 2. Epilepsy Behav. EB.

[20] Wu Y., Zhang Z., Dong X., Hong S., Hu Y., Liang P., Li L., Zou B., Wu X., Wang D. (2024). Evaluating the performance of the language model ChatGPT in responding to common questions of people with epilepsy. Epilepsy Behav. EB.

[21] Kim H.W., Shin D.H., Kim J., Lee G.H., Cho J.W. (2024). Assessing the performance of ChatGPT’s responses to questions related to epilepsy: A cross-sectional study on natural language processing and medical information retrieval. Seizure.

[22] Mauritz M., Hirsch L.J., Camfield P., Chin R., Nardone R., Lattanzi S., Trinka E. (2022). Acute symptomatic seizures: An educational, evidence-based review. Epileptic Disord..

